# How long can you hold on? Physical self-efficacy predicts performance estimation accuracy independent of leisure-time physical activity

**DOI:** 10.3389/fpsyg.2025.1545582

**Published:** 2025-04-25

**Authors:** Friedrich Meixner, Sophia Wölfle, Nizar Hawat

**Affiliations:** ^1^Department of Psychology, Macromedia University of Applied Sciences, Stuttgart, Germany; ^2^Independent Researcher, Ulm, Germany

**Keywords:** physical self-efficacy, performance estimation accuracy, physical activity, self-regulation, psychophysiological factors, sports performance, self-assessment

## Abstract

**Introduction:**

Accurately estimating future performance is crucial for optimizing performance in sports and exercise. In our study, we aimed to explore the relationship between physical self-efficacy and the accuracy of performance estimation in various physical exercises.

**Methods:**

Data were collected from *N* = 31 students (*M* = 23.5 years, *n* = 23 female, BMI 17–30, not engaged in any competitive sports). Measurements included questionnaires on physical activity and physical self-efficacy. Participants estimate their performance in five exercises, prior to performing them: (a) dumbbell hold, (b) plank, (c) vertical jump, (d) grip strength and (e) flamingo balance test.

**Results:**

Independently of leisure-time physical activity, participants underestimated their performance in these exercises. Physical self-efficacy was neither associated with levels of intense, leisure-time physical activity (*r* = 0.243, *p* > 0.05), nor with objective performance (all *p* > 0.05). However, physical self-efficacy was significantly associated with greater accuracy in performance estimation across all exercises (*p* < 0.01). These relationships were not mediated by leisure-time physical activity.

**Conclusion:**

Physical self-efficacy was positively associated with the congruency between estimation and objective performance, independent of leisure-time physical activity. These findings contribute to self-regulation research by emphasizing self-efficacy as a key factor in performance estimation accuracy, prioritizing cognitive mechanisms over behavioral engagement in self-assessment, and highlight its potential relevance in coaching and self-regulation.

## Introduction

Accurately forecasting one’s physical performance, i.e., estimating personal performance in a given task, is an essential factor in avoiding under- or overexertion in exercise, sports and competition. Performance estimation accuracy describes the congruency between an individual’s subjective performance capabilities and subsequent, actual performance outcomes. Understanding the factors contributing to this accuracy is crucial for optimizing self- and autoregulation processes in physical activities, e.g., pacing in endurance and strength endurance sports, selecting appropriate weights during resistance training, or, for example, conservation of energy in combat sports. Recent meta-analytical findings suggest that self-regulation is a critical component in sports psychology, influencing both cognitive and behavioral engagement (e.g., McAuley et al., 2005; Szczuka et al., 2021). These models highlight the interplay between self-efficacy, outcome expectations, and self-regulation, reinforcing the need to examine how self-efficacy contributes to performance estimation accuracy.

Previous research has investigated potential factors explaining interindividual differences in performance estimation. Halperin et al. (2022) found that, with notable heterogeneity, participants generally underpredicted their repetitions until task failure in the given exercise was reached (Steele et al., 2017). Factors influencing performance prediction have been hypothesized to be exercise modality, i.e., lower- vs. upper body exercises (Remmert et al., 2023), fatigue (Armes et al., 2020) and training experience (Steele et al., 2017). Additionally, cognitive and metacognitive processes, such as physical self-efficacy, are believed to contribute to these differences (Horcajo et al., 2022).

Physical self-efficacy is defined as an individual’s belief in their capacity to successfully execute specific tasks (Bandura, 1997, 2001). In extension, physical self-efficacy has proven to be a significant predictor of regular physical activity, improved motor performance, and enhanced overall well-being (Hagger et al., 2001; Higgins et al., 2014; Koeneman et al., 2011; McAuley et al., 2005), as increased physical self-efficacy or perceived physical competence increases the likelihood to engage in physical activity (Self-Determination Theory; Ryan and Deci, 2000). Recent meta-analyses further support this link—Szczuka et al. (2021) found that higher self-efficacy was associated with reduced sedentary behavior (*r* = −0.158), indicating its role in promoting an active lifestyle across age groups. These findings provide additional context for how self-efficacy influences engagement in physical activity. Beyond engagement in physical activity, recent studies have also examined strategies for effectively increasing self-efficacy. A systematic review by Tang et al. (2019) identified behavior change techniques (BCTs) that contribute to both immediate and sustained improvements in self-efficacy for physical activity. Their meta-analysis found small but significant effects (*d* = 0.26) for post-intervention self-efficacy, suggesting that self-efficacy is malleable and can be enhanced through targeted interventions.

Despite the well-established role of physical self-efficacy in self- and autoregulation in sports and exercise (Horcajo et al., 2022) and its potential contribution to physical activity planning (Szczuka et al., 2023) its impact on performance estimation accuracy, particularly in relation to physical activity levels, remains underexplored. Given that both planning physical activity and estimating performance possibly rely on predictive cognitive mechanisms, including self-referential thought, interoceptive awareness, and experience, it is plausible that self-efficacy not only shapes behavioral engagement but also influences an individual’s ability to accurately project their capabilities onto future physical tasks. Our study aims to evaluate the effects of physical self-efficacy and engagement in leisure-time physical activity on performance estimation accuracy across multiple exercise tasks.

Hypotheses for our study are based on previous research literature suggesting a strong relationship between physical self-efficacy and higher physical activity levels (e.g., Devereux-Fitzgerald et al., 2016; Hagger et al., 2001; Higgins et al., 2014; Koeneman et al., 2011; McAuley et al., 2005). While self-efficacy has been extensively studied as a predictor of physical activity engagement, its role in performance estimation accuracy, as well as the potential mediating effect of leisure-time physical activity on this relationship, remain underexplored—leaving a gap in understanding how habitual movement experiences shape self-assessment processes in physical performance.

Firstly, it is expected that individuals with higher levels of physical self-efficacy will engage more frequently in leisure-time physical activity, as self-efficacy has been frequently shown to be a predictor of behavioral engagement across multiple contexts (e.g., Devereux-Fitzgerald et al., 2016; Hagger et al., 2001; Higgins et al., 2014; Koeneman et al., 2011; McAuley et al., 2005).

*H*1: Higher physical self-efficacy is associated with more frequent engagement in leisure-time physical activity.

Secondly, Individuals who frequently engage in physical activity, or who have greater self-efficacy in physical tasks, are more likely to exert effort when facing physical challenges.

*H*2: Higher physical self-efficacy and greater leisure-time physical activity are both associated with better physical performance outcomes.

Lastly, it is hypothesized that individuals with high self-efficacy may be better at assessing their actual physical capabilities due to heightened self-awareness and confidence in their abilities. Additionally, frequent engagement in physical activity may refine one’s ability to accurately estimate performance.

*H*3: Higher levels of self-efficacy lead to increased congruency between subjective performance estimates and objective performance measures, improving performance estimation accuracy.

*H*3a: This relationship is expected to be mediated by leisure-time physical activity.

## Methods

### Recruitment and participants

Participants were recruited at Macromedia University of Applied Sciences Stuttgart via posters and in-class announcements. Participants were eligible if they were at least 18 years old, had a BMI between 17 and 30, and did not suffer from any physical disabilities or chronic health conditions. To maintain a homogeneous sample, participants actively engaged in competitive, performance-oriented sports were excluded to prevent potential confounding effects of advanced training experience on performance estimation accuracy. The final sample consisted of 31 participants (*M* = 23.45 years, *SD* = 9.31, *n* = 23 female participants). Most participants (90.3%) were psychology students, with 3.2% in vocational training and 6.5% University employees. Participants were not engaged in competitive sports but reported intense leisure-time physical activity (e.g., working out) for *M* = 175 (*SD* = 235) minutes per week. All participants were informed about the goals of this study and provided written informed consent before participation. They were explicitly informed that participation was voluntary, that they could withdraw at any time without consequences, and that their data would be anonymized to ensure confidentiality. Compensation was provided in the form of course credit, following university guidelines. The study was conducted in accordance with the ethical standards of the Declaration of Helsinki. A formal ethics review was not required, as confirmed by our institutional review board, which determined that the study did not fall under categories necessitating a full ethics review.

Although the sample size in our study (*N* = 31) is relatively small due to feasibility constraints, it is comparable to sample sizes used in prior research on performance estimation accuracy. Several studies included in Halperin et al. (2022) review and meta-analysis on the accuracy of predicting repetitions to failure in resistance training featured similar or smaller participant numbers, demonstrating that meaningful effects can be detected within this range. A *post hoc* power analysis using G*Power for a linear regression model (one predictor, *α* = 0.05) indicated that *N* = 31 provides >80% power to detect a large effect size (*f*^2^ > 0.3).

### Study design and measures

In a cross-sectional design, we collected quantitative data on self-efficacy, regular physical activity at work and in leisure time, performance estimations and lastly, objective performance parameters. Physical self-efficacy has been measured using a German version of the Physical Self-Efficacy Questionnaire (Ryckman et al., 1982; translated by the authors).

Physical activity levels were assessed using the Global Physical Activity Questionnaire (GPAQ; Armstrong and Bull, 2006). Participants reported the frequency and duration of moderate and intense physical activities in leisure time or during their commutes and homework performed in a typical week. For the subsequent analyses, we focused specifically on intense leisure-time physical activity, as it most closely aligns with the demands of the evaluated exercises.

Performance estimation accuracy was operationalized as the absolute difference between participants’ subjective performance estimates and objective performance outcomes. Exercises have been selected to ensure full-body utilization, simplicity, to include dynamic and static components, and have been loosely based on the EuroFit Test Battery, a standardized test battery for physical capabilities (Grgic, 2023; Tsigilis et al., 2002). This selection was intended to provide a balance between ecological validity and standardization, making the tasks accessible to all participants while maintaining structured measurement criteria. Participants estimated their performance in five exercises before performing each task. Objective measures were recorded by the experimenter to assess the accuracy of participants’ performance estimations.

### Procedure

Upon arrival in the laboratory, participants completed the GPAQ and Physical Self-Efficacy Questionnaire via an online platform (Sosci Survey). After completing the questionnaires, participants were then asked to remove their shoes, and a short warm-up session was instructed by the experimenter. Participants were subsequently instructed on the procedure for the first exercise, after which the first exercise was estimated and immediately performed. Upon completion, participants were instructed on the procedure for the second exercise etc. Subjective estimates and objective measures were recorded for all exercises. Sequencing of the exercises has been randomized for each participant.

Before each exercise, participants were allowed a brief 3–5 s familiarization period and were instructed on proper technique until satisfactory and safe execution was achieved. They were asked to estimate their future performance in the given task, if they utilized maximum effort. This brief familiarization period was chosen to ensure task comprehension while minimizing potential learning effects that could influence performance predictions. Longer familiarization might have led to unintended adaptation, where participants refine their estimates based on real-time adjustments rather than relying on pre-existing self-assessment abilities.

The tasks included: (a) Dumbbell Hold, where participants held a 5 lbs. dumbbell straight out in front of them, in a straight line from shoulder to hand, thumb pointing upwards, shoulders kept down, with the corresponding trapezius muscle as relaxed as possible, (b) a Plank position on a padded surface, with only their feet, elbows, and hands touching the floor, elbows and shoulders in a vertical line, while maintaining a straight back, (c) a Vertical Jump from a standing position, where participants jumped straight up as high as possible, with a pen in one hand, the corresponding arm extended upwards to mark their jump height on a nearby wall poster, (d) a test of Grip Strength, where participants squeezed a hand dynamometer to get a feeling for their maximum grip strength. Regarding performance estimation, they were asked to estimate how long they could maintain 60% of that grip strength. This test ended when their grip strength fell below −10% of the latter value. The last exercise was (e) the Flamingo Balance test, where participants had to stand on one foot while holding the other foot in one hand and estimate how many times they would lose balance over a 60-s period.

For each task, participants provided time estimates for how long they could maintain the position/force output (dumbbell hold, plank, grip strength), their expected jump height (vertical jump), or anticipated balance losses (flamingo balance).

### Data analysis

Data were analyzed using SPSS 29 and Jamovi. Descriptive statistics were computed to assess the distribution of demographic and psychometric variables. Performance estimation accuracy was calculated as the difference scores between estimated and actual performance. Aside from descriptive analyses of under- vs. overestimation, we focused on accuracy regardless of underestimation or overestimation. Therefore, only absolute differences have been used in subsequent analyses.

Correlational and linear regression analyses have been performed to test the relationships between physical self-efficacy (PSE), intense leisure-time physical activity, and the accuracy of performance estimation. Mediation analyses were performed to examine whether intense leisure-time physical activity mediated the relationship between self-efficacy and estimation accuracy. A 1000 sample bootstrap procedure has been applied to calculate the indirect, direct, and total effects.

## Results

Descriptive analyses revealed that participants generally underestimated their performance across all physical tasks, with mean absolute differences between subjective estimates and objective outcomes significantly greater than zero (all *p* < 0.01). This indicates a general tendency toward underestimation among participants, which is in line with results from the literature (Halperin et al., 2022). This has been especially visible in the Flamingo Balance Task, where no participant lost balance during the required 60s. Due to this ceiling effect, this exercise has been excluded from further analyses.

Hypothesis 1, which proposed a positive relationship between physical self-efficacy (PSE) and leisure-time activity, was not supported. The relationship between PSE and levels of leisure-time physical activity has been positive, but non-significant (*r* = 0.24, *p* > 0.05, 95% CI [−0.12, 0.55]), indicating that self-efficacy did not predict engagement in leisure-time physical activity in our sample.

Moreover, Hypothesis 2 proposed the association of higher self-efficacy with increased, objective physical performance, i.e., increased physical capability. However, no significant relationship between PSE and performance outcomes could be shown in any of the four exercises a-d (all *p* > 0.05).

**Figure 1:**
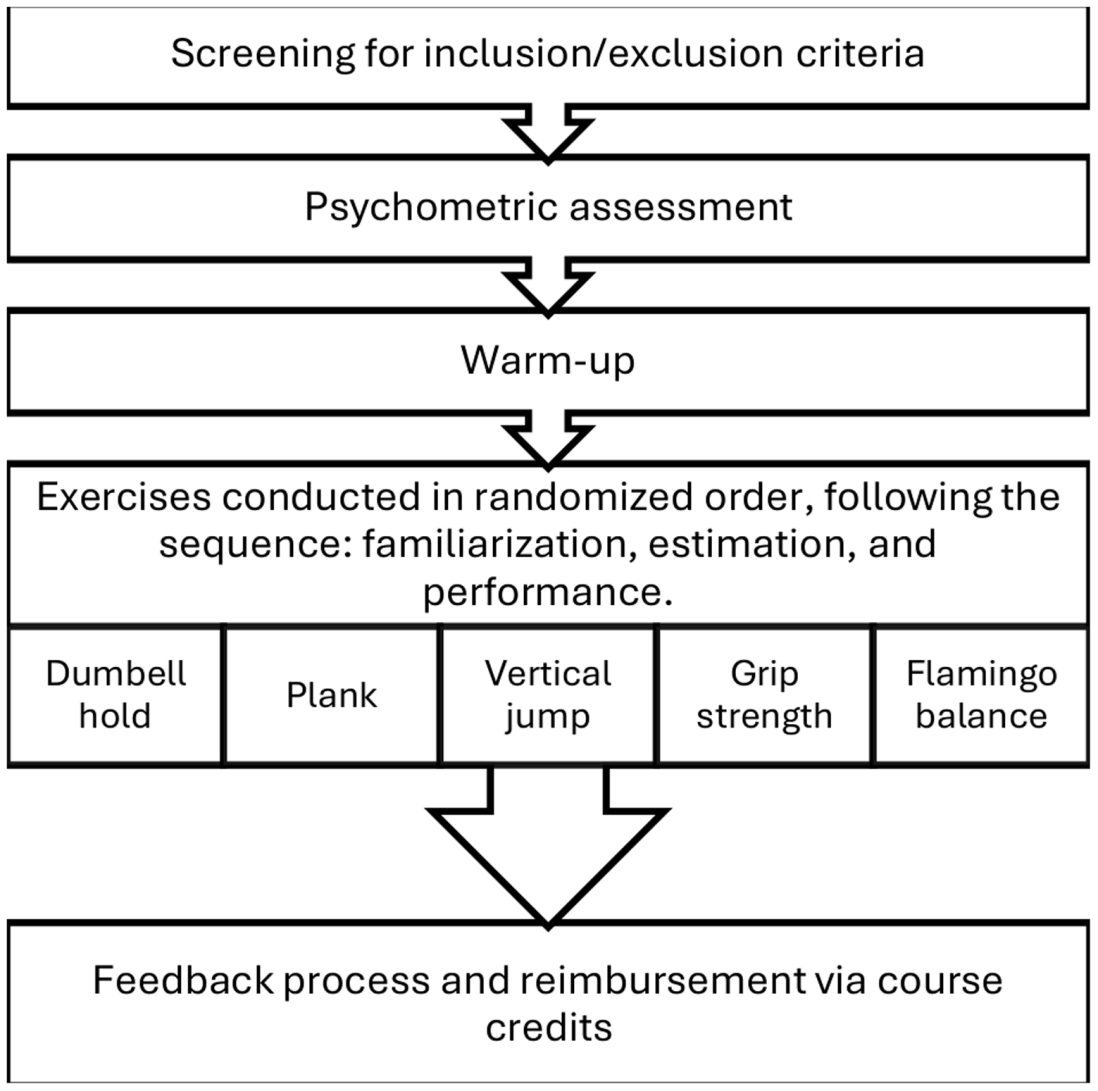
Flowchart illustrating the 30-min experimental procedure.

Lastly, our data supported Hypothesis 3, as higher PSE was associated with a greater congruence between subjective estimates and objective performance outcomes (all *p* < 0.01). Linear regression models confirmed that PSE significantly predicted estimation accuracy for all tasks (see Table 1 and Figure 1). Across all four exercises a-d, higher PSE was consistently associated with greater congruency (i.e., reduced estimation error) and explained 24.7–36.1% of the variance in performance estimation accuracy.

**Table 1:** Regression analyses predicting performance estimation accuracy across exercises from physical self-efficacy.

Task	B	SE	*β*	*t*	*p*	95% CI	R^2^
Dumbbell Hold	−0.75	0.19	−0.586	−3.90	<0.001	[−0.894, −0.279]	0.344
Plank Hold	−0.71	0.18	−0.590	−3.94	<0.001	[−0.897, −0.284]	0.348
Standing High Jump	-0.28	0.09	−0.522	−3.29	0.003	[−0.846, −0.198]	0.272
Grip Strength	−0.58	0.14	−0.601	−4.05	<0.001	[−0.905, −0.297]	0.361

Results of separate linear regression analyses predicting performance estimation accuracy for each exercise based on physical self-efficacy (PSE). Higher PSE was significantly associated with reduced discrepancy in all tasks (*p* < 0.01), indicating that individuals with higher self-efficacy exhibited higher accuracy in estimating their own future performance. Unstandardized coefficients (B), standard errors (SE), standardized regression weights (β), *t*-values, significance levels (*p*), 95% confidence intervals (CI), and model fit indices (R^2^) are reported.

**Figure 2:**
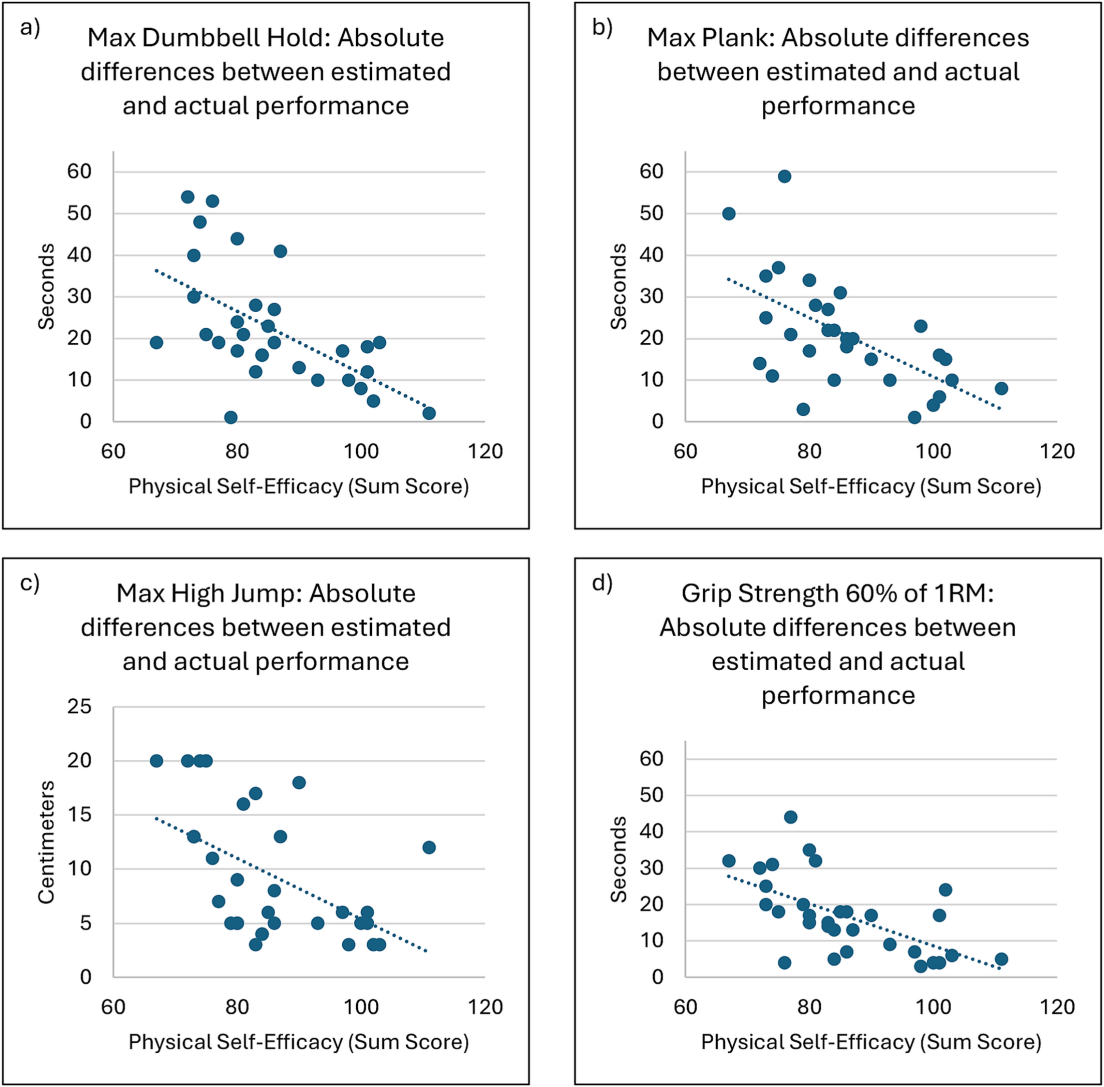
Scatterplots of linear regression models showing the relationship between physical self-efficacy (PSE) and performance estimation accuracy across all exercises. Each panel corresponds to one exercise: dumbbell hold **(a)**, plank hold **(b)**, standing high jump **(c)**, and grip strength **(d)**. Higher PSE was significantly associated with greater estimation accuracy in all exercises (*p* < 0.01), indicating that individuals with higher self-efficacy exhibited a smaller discrepancy between estimated and actual performance. Regression statistics, including effect sizes and confidence intervals, are detailed in Table 1.

For the dumbbell hold exercise (Figure 2a), the regression model revealed a significant negative relationship between self-efficacy and estimation error (*β* = −0.586, *p* < 0.001). The model accounted for a moderate proportion of variance in estimation accuracy (*R^2^* = 0.344). These results indicate that physical self-efficacy explains approximately 34.4% of the variance in estimation accuracy for this task.

Similar findings emerged for the plank hold exercise (Figure 2b), where self-efficacy was also a significant negative predictor of estimation error (*β =* −0.590*, p* < 0.001). 34.8% of the variance could be explained by the model (*R^2^ =* 0.348). Assumptions of linearity, normal distribution of residuals, and homoscedasticity were met. However, two outliers were identified, which may have influenced the model fit.

The standing high jump exercise (Figure 2c) similarly demonstrated a significant negative relationship between self-efficacy and estimation error (*β* = −0.522, *p* = 0.003). The model explained 27.2% of the variance in estimation accuracy for this task (*R^2^* = 0.272). While the linearity assumption was satisfied, the Durbin-Watson test for autocorrelation indicated some level of autocorrelation (1.43), albeit not significant (*p* > 0.05) and the residuals did not follow a normal distribution (*p* = 0.024). However, due to our sample size, it can be assumed that the given parameter estimates, and significance tests remain robust to these violations.

Finally, for the grip strength exercise (Figure 2d), self-efficacy significantly predicted estimation accuracy (*β =* −0.601, *p* < 0.001), and explained 36.1% of the variance (*R^2^* = 0.361). Although the assumptions of linearity, homoscedasticity, and absence of outliers were met, the Durbin-Watson statistic (1.33) indicated a potential issue with autocorrelation, which may have affected the validity of the model. Therefore, the latter result needs to be interpreted accordingly. However, taken together, the findings confirm that higher self-efficacy is consistently associated with lower estimation error across tasks.

Next, mediation analyses using 1,000 bootstrap samples were conducted to analyze whether intense leisure-time physical activity mediated the relationship between PSE and performance estimation accuracy.

Results demonstrate that intense leisure-time physical activity did not significantly mediate this relationship in any of the exercises. Indirect effects were small and non-significant (a) Dumbbell Hold: 0.0936, 95% CI [−0.104, 0.225], *p* = 0.241 (10.0% mediated), (b) Plank: 0.139, 95% CI [−0.0961, 0.301], *p* = 0.156 (14.1% mediated), (c) Vertical Jump: −0.0156, 95% CI [−0.135, 0.0213], *p* = 0.695 (5.53% mediated) and (d) Grip Strength: −0.0807, 95% CI [−0.181, 0.0876], *p* = 0.226 (13.9% mediated). The mediation models also provided detailed path estimates for direct and indirect relationships:

Path a (PSE → Intense Leisure-Time Physical Activity): Across all tasks, self-efficacy was positively associated with intense physical activity, though these relationships were not statistically significant (all *p* > 0.05).

Path b (Intense Leisure-Time Physical Activity → Performance Estimation Accuracy): Intense physical activity showed small, occasionally significant effects on estimation accuracy for certain tasks, such as dumbbell hold (*β* = 0.00227, *p* = 0.037) and plank hold (*β* = 0.00338, *p* < 0.001).

Path c’ (Direct Effect of PSE → Performance Estimation Accuracy): The direct effects of self-efficacy on estimation accuracy were consistently strong and significant (all *p* < 0.001), highlighting its central role in reducing discrepancies between perceived and actual performance. These direct effects accounted for 85.9 to 94.5% of the total effect across tasks.

## Discussion

While previous research has emphasized the relationship between self-efficacy and physical activity (e.g., Devereux-Fitzgerald et al., 2016; Hagger et al., 2001; Higgins et al., 2014; Koeneman et al., 2011; McAuley et al., 2005), our study suggests that self-efficacy’s impact on performance estimation accuracy seems to work independently of increased physical activity levels. Therefore, our results point toward performance estimation accuracy being more closely tied to the cognitive processes regarding physical self-efficacy.

The cognitive processes underlying self-efficacy have been widely discussed in the literature, emphasizing its role in cognitive appraisal, effort regulation, and behavioral persistence (Bandura, 1997; Horcajo et al., 2022; Szczuka et al., 2021, 2023). Our findings support this by demonstrating that individuals with higher self-efficacy tend to exhibit greater accuracy in self-assessment, regardless of their physical activity levels. This suggests that self-efficacy may enhance performance estimation through mechanisms such as improved attentional focus, better recognition of physical exertion cues, or refined predictive abilities regarding task difficulty.

Additionally, meta-analytical findings suggest that physical self-efficacy not only influences engagement in physical activity but also plays a role in the anticipation and planning of future physical activities, at least in a broader sense (cultivation hypothesis; Szczuka et al., 2021, 2023). Given that self-efficacy has been linked to reduced sedentary behavior and increased motivation for physical engagement, it is therefore likely that self-efficacy plays more than one role by (a) facilitating behavioral engagement, while also (b) simultaneously enhancing cognitive self-monitoring processes that could contribute to more accurate performance estimations.

Relating to this notion of multiple functions, leisure-time physical activity did not mediate the relationship between self-efficacy and estimation accuracy. To better understand this finding, it is important to first examine the direct association between self-efficacy and physical activity. While the observed correlation (*r* = 0.24) was consistent with previous findings (McAuley et al., 2005), it did not reach statistical significance, likely due to our limited sample size (*N* = 31). This raises the question of whether insufficient statistical power contributed to the non-significant mediation. As results from our mediation analysis indicate, indirect effects were small and non-significant across all tasks, with no trend toward mediation. The strong direct effects of self-efficacy on estimation accuracy (all *p* < 0.001) suggest that self-efficacy primarily influences cognitive self-monitoring and attentional control mechanisms, rather than influencing estimation accuracy through increased engagement in physical activity. This reinforces the idea that performance estimation accuracy is primarily shaped by cognitive rather than behavioral mechanisms, such as attentional focus (Meixner and Herbert, 2021, 2022) or metacognitive processes (Horcajo et al., 2022).

Another possible explanation for the non-significant correlation between PSE and physical activity lies in the measurement approach, as self-reported activity data have well-documented limitations in accurately capturing intensity, frequency and volume in a retrospective fashion. Additionally, our sample consisted primarily of students, many of whom engaged in only one type of leisure-time sport or none at all, which may have weakened the association between broad, task-unspecific self-efficacy measures and actual physical activity levels. Future studies should incorporate objective measures, such as accelerometers or wearable devices, and consider more domain-specific self-efficacy assessments to better evaluate the relationship between self-efficacy and physical activity.

Taken together, our findings suggest that novices cannot be expected to develop accurate performance estimation “on the go” simply by being more active. Rather than assuming self-assessment automatically improves by frequent training, our results rather point toward working on self-efficacy (see Tang et al., 2019) to improve estimation accuracy. Indirectly, as self-efficacy seems to be associated with the planning component of future physical activity (Szczuka et al., 2021, 2023), cognition-based and metacognition-based strategies (see also Horcajo et al., 2022), such as guided self-reflection on performance, mental rehearsal, and structured performance feedback, could also prove to be effective means in teaching young adults how to assess their physical capabilities. For example, mental rehearsal techniques could be incorporated into training regimes to improve the assessment of future tasks’ difficulty and demand more accurately, which is likely to be the foundation for performance estimation, and in turn, self-regulation or pacing. These approaches are especially relevant in sports requiring precise self-assessment for performance regulation, such as endurance sports or weightlifting, where misjudging one’s abilities can lead to overtraining, early burnout via suboptimal pacing or underperformance.

Furthermore, our findings also align with Wulf and Lewthwaite’s (2016) OPTIMAL Theory of Motor Learning, which highlights the role of motivational factors in enhancing motor learning. Specifically, enhanced expectations for success contribute to more efficient skill acquisition by strengthening goal-action coupling. Self-efficacy may be particularly relevant in this framework, as it is closely tied to expectancy-based motivation, reinforcing confidence in one’s ability to succeed in a task. This also underlines the importance of self-efficacy in coaching and training young athletes not only for optimal performance, but also for optimal skill acquisition.

### Limitations

The major limitation of our study is the small, homogenous sample size, limiting the generalizability of the findings. For the most part, our participants were young, female, untrained individuals, making it difficult to draw conclusions that apply to, e.g., male, or trained individuals or older populations.

Another key limitation concerns the complexity of the exercises used in this study. The standing high jump, for example, involves multiple components, such as coordination, lower-body strength, and balance, making it inherently more challenging to estimate accurately compared to simpler, more static tasks such as a grip strength test using a hand dynamometer. Similarly, muscle endurance tasks, such as the dumbbell hold, while simple, are rarely performed in everyday life, potentially leading to greater uncertainty in performance estimation despite the short familiarization. A related methodological limitation is the brief familiarization period before task execution. While this prevented learning effects from influencing performance estimation, it may have increased variability in prediction accuracy, particularly for more complex or unfamiliar exercises. Participants with less prior experience may have been at a disadvantage in making accurate predictions, which could have influenced the strength of observed relationships.

Our regression analyses indicated potential issues with outliers and autocorrelation in some models. The plank and standing high jump models contained outliers, while the grip strength and standing high jump regressions showed some signs of autocorrelation. While no extreme outliers exceeded standard thresholds (±3 SD), their presence may have inflated variance estimates, potentially impacting the precision of regression coefficients. Similarly, autocorrelation could have affected the robustness of significance testing. However, given our study’s exploratory nature, we retained all data points to maximize statistical power.

As highlighted by Halperin et al. (2022), timing of predictions and additional task-specific factors besides complexity may also influence estimation accuracy, as predictions made closer to task failure or during later sets seem to be generally more accurate. However, in our study, the exercises were designed to be performed in single sets, after one submaximal warm-up set. Our exercises also included one-repetition tasks, such as the high jump, or time-based tasks, such as the plank hold or dumbbell hold, where participants managed to sustain the position for 60+ seconds. For these participants, our time-based tasks extended beyond the duration typically required to complete, e.g., 8–12 repetitions. This might have introduced additional challenges to performance estimation accuracy, which would be unrelated to physical fitness, but rather to numerical estimation biases (Halperin et al., 2022).

To address these limitations, future research should aim to increase sample diversity and examine whether experience, sex differences, or age influence the relationship between self-efficacy and performance estimation accuracy. Additionally, further investigation is needed to determine how movement complexity and familiarity shape estimation accuracy by systematically varying task difficulty, familiarization, and repetition structure. Extended familiarization or repeated exposure may improve self-assessment accuracy, particularly for complex or unfamiliar tasks. Also, incorporating objective physical activity measures, such as wearables and accelerometers, could refine data collection and minimize recall bias in future studies. Finally, future research projects should consider how different time frames and repetition ranges affect self-assessment, providing deeper insights into how self-efficacy influences performance estimation across different physical tasks.

## Conclusion

Our study underlines the critical role of physical self-efficacy for performance estimation accuracy across multiple exercise domains. The results indicate that physical self-efficacy, rather than leisure-time physical activity, is the primary factor explaining the accuracy of performance estimates, i.e., the discrepancy between subjective estimation and objective performance. This provides a valuable addition to existing literature, underlining the importance of physical self-efficacy for self-regulation, and therefore effective training or competitive strategies.

Given the practical implications for coaching and teaching, our findings suggest that higher self-efficacy is associated with greater performance estimation accuracy. While our study does not establish causality, targeted self-efficacy interventions, such as performance feedback and metacognitive reflection strategies, could improve self-assessment accuracy and adaptive decision-making in physical performance.

## Data Availability

The raw data supporting the conclusions of this article will be made available by the authors, without undue reservation.

## References

[ref1] Armes C. Standish-Hunt H. Androulakis-Korakakis P. Michalopoulos N. Georgieva T. Hammond A.. (2020). “Just one more rep!” – ability to predict proximity to task failure in resistance trained persons. Front. Psychol. 11:565416. doi: 10.3389/fpsyg.2020.565416, PMID: 33424678 PMC7785525

[ref2] Armstrong T. Bull F. (2006). Development of the World Health Organization global physical activity questionnaire (GPAQ). J. Public Health 14, 66–70. doi: 10.1007/s10389-006-0024-x

[ref3] Bandura A. (1997). Self-efficacy: The exercise of control, vol. 43. New York: W.H Freeman and Company.

[ref9001] Bandura A. (2001). Social cognitive theory: An agentic perspective. Annu. Rev. Psychol. 52, 1–26. doi: 10.1146/annurev.psych.52.1.1 11148297

[ref4] Devereux-Fitzgerald A. Powell R. Dewhurst A. French D. P. (2016). The acceptability of physical activity interventions to older adults: a systematic review and meta-synthesis. Soc. Sci. Med. 158, 14–23. doi: 10.1016/j.socscimed.2016.04.006, PMID: 27104307

[ref5] Grgic J. (2023). Test–retest reliability of the EUROFIT test battery: a review. Sport Sci. Health 19, 381–388. doi: 10.1007/s11332-022-00936-x

[ref6] Hagger M. S. Chatzisarantis N. Biddle S. J. H. (2001). The influence of self-efficacy and past behaviour on the physical activity intentions of young people. J. Sports Sci. 19, 711–725. doi: 10.1080/02640410152475847, PMID: 11522147

[ref7] Halperin I. Malleron T. Har-Nir I. Androulakis-Korakakis P. Wolf M. Fisher J.. (2022). Accuracy in predicting repetitions to task failure in resistance exercise: a scoping review and exploratory meta-analysis. Sports Med. 52, 377–390. doi: 10.1007/s40279-021-01559-x, PMID: 34542869

[ref8] Higgins T. J. Middleton K. R. Winner L. Janelle C. M. (2014). Physical activity interventions differentially affect exercise task and barrier self-efficacy: a meta-analysis. Health Psychol. 33, 891–903. doi: 10.1037/a0033864, PMID: 23957904 PMC4148031

[ref9] Horcajo J. Santos D. Higuero G. (2022). The effects of self-efficacy on physical and cognitive performance: an analysis of meta-certainty. Psychol. Sport Exerc. 58:102063. doi: 10.1016/j.psychsport.2021.102063

[ref10] Koeneman M. A. Verheijden M. W. Chinapaw M. J. M. Hopman-Rock M. (2011). Determinants of physical activity and exercise in healthy older adults: a systematic review. Int. J. Behav. Nutr. Phys. Act. 8:142. doi: 10.1186/1479-5868-8-142, PMID: 22204444 PMC3320564

[ref11] McAuley E. Elavsky S. Motl R. W. Konopack J. F. Hu L. Marquez D. X. (2005). Physical activity, self-efficacy, and self-esteem: longitudinal relationships in older adults. J. Gerontol. B Psychol. Sci. Soc. Sci. 60, P268–P275. doi: 10.1093/geronb/60.5.P268, PMID: 16131621

[ref12] Meixner F. Herbert C. (2021). Does Attentional Focus Influence Psychophysiological Responses to an Acute Bout of Exercise? Evidence From an Experimental Study Using a Repeated-Measures Design. Front. Physiol. 12:829. doi: 10.3389/FPHYS.2021.680149/BIBTEX PMC8267581 34248667

[ref13] Meixner F. Herbert C. (2022). Acute aerobic exercise and attentional focus influence the self-positivity bias in emotional evaluation. Evidence from an experimental study. Open Psychol. 4, 187–204. doi: 10.1515/PSYCH-2022-0010

[ref14] Remmert J. F. Laurson K. R. Zourdos M. C. (2023). Accuracy of predicted Intraset repetitions in reserve (RIR) in single- and multi-joint resistance exercises among trained and untrained men and women. Percept. Mot. Skills 130, 1239–1254. doi: 10.1177/00315125231169868, PMID: 37036795

[ref9002] Ryan R. M. Deci E. L. (2000). Self-Determination Theory and the Facilitation of Intrinsic Motivation, Social Development, and Well-Being. Am Psychol. 55, 68–78. doi: 10.1037/0003-066X.55.1.68 11392867

[ref15] Ryckman R. M. Robbins M. A. Thornton B. Cantrell P. (1982). Development and validation of a physical self-efficacy scale. J. Pers. Soc. Psychol. 42, 891–900. doi: 10.1037/0022-3514.42.5.891

[ref16] Steele J. Endres A. Fisher J. Gentil P. Giessing J. (2017). Ability to predict repetitions to momentary failure is not perfectly accurate, though improves with resistance training experience. PeerJ 5:e4105. doi: 10.7717/peerj.4105, PMID: 29204323 PMC5712461

[ref17] Szczuka Z. Banik A. Abraham C. Kulis E. Luszczynska A. (2021). Associations between self-efficacy and sedentary behaviour: a meta-analysis. Psychol. Health 36, 271–289. doi: 10.1080/08870446.2020.1784419, PMID: 32597242

[ref18] Szczuka Z. Kulis E. Boberska M. Banik A. Siwa M. Zaleskiewicz H.. (2023). Dyadic reciprocal associations between self-efficacy and planning predict sedentary behaviour. Br. J. Health Psychol. 28, 451–466. doi: 10.1111/bjhp.12633, PMID: 36333942

[ref19] Tang M. Y. Smith D. M. Mc Sharry J. Hann M. French D. P. (2019). Behavior change techniques associated with changes in postintervention and maintained changes in self-efficacy for physical activity: a systematic review with meta-analysis. Ann. Behav. Med. 53, 801–815. doi: 10.1093/abm/kay090 30534971

[ref20] Tsigilis N. Douda H. Tokmakidis S. P. (2002). Test-retest reliability of the eurofit test battery administered to university students. Percept. Mot. Skills 95, 1295–1300. doi: 10.2466/pms.2002.95.3f.1295 12578274

[ref21] Wulf G. Lewthwaite R. (2016). Optimizing performance through intrinsic motivation and attention for learning: the OPTIMAL theory of motor learning. Psychon. Bull. Rev. 23, 1382–1414. doi: 10.3758/s13423-015-0999-9, PMID: 26833314

